# Association of Systemic Inflammatory Indices with Metabolic Dysfunction–Associated Steatotic Liver Disease and Liver Fibrosis

**DOI:** 10.5152/tjg.2026.25656

**Published:** 2026-02-12

**Authors:** Qinghua Wei, Neng Shen, Xiang Chen, Jiacheng Cai

**Affiliations:** The Third People’s Hospital of Yinzhou District, Ningbo, Zhejiang, People’s Republic of China

**Keywords:** CAP, liver fibrosis, LSM, MASLD, NHANES, systemic inflammation index

## Abstract

**Background/Aims:**

The newly proposed metabolic dysfunction–associated steatotic liver disease (MASLD) framework reflects the evolving understanding of fatty liver disease, highlighting the importance of exploring novel risk factors beyond traditional metabolic indicators. This study aimed to examine the associations between systemic inflammatory indices and the prevalence of MASLD and liver fibrosis.

**Materials and Methods:**

**:** Data from the 2017 MASLD framework reflecting the evolving regression was used to assess associations of systemic inflammatory indices—systemic immune-inflammation index (SII), systemic inflammation response index (SIRI), platelet-to-lymphocyte ratio (PLR), lymphocyte-to-monocyte ratio (LMR), prognostic nutritional index (PNI), aggregate index of systemic inflammation (AISI), inflammation burden index (IBI), neutrophil-to-lymphocyte ratio, neutrophil-to-albumin ratio, and pan-immune-inflammation value (PIV)—with MASLD assessed by controlled attenuation parameter (CAP) and liver fibrosis assessed by liver stiffness measurement (LSM), adjusting for confounders.

**Results:**

Analysis of CAP and MASLD revealed that elevated SII, SIRI, LMR, IBI, and PNI were significantly associated with higher CAP values, reflecting increased hepatic steatosis, while PLR showed a negative association. Regarding LSM and liver fibrosis, higher SIRI, PNI, IBI, and AISI were significantly associated with increased liver stiffness, whereas PLR remained inversely correlated.

**Conclusion:**

This nationally representative study demonstrates that systemic inflammatory dysregulation is linked to both MASLD and liver fibrosis, with distinct biomarker patterns for steatosis and fibrosis.

**Main Points**

- Associations between systemic inflammatory biomarkers and liver health were evaluated, with controlled attenuation parameter reflecting hepatic steatosis and liver stiffness measurement reflecting liver fibrosis.
- Elevated inflammatory indices were linked to higher odds of metabolic dysfunction–associated steatotic liver disease.
- This study provides novel evidence on the relationship between composite systemic inflammatory indices and liver fibrosis, an area previously unexplored in population-based cohorts.

## Introduction

Nonalcoholic fatty liver disease (NAFLD) has emerged over the past 3 decades as the leading cause of chronic liver disease worldwide, paralleling the global epidemics of obesity and type 2 diabetes mellitus (T2DM).^1^ Defined by the accumulation of hepatic steatosis in the absence of significant alcohol consumption or other specific etiologies, NAFLD encompasses a broad histological spectrum ranging from simple steatosis to nonalcoholic steatohepatitis, advanced fibrosis, and cirrhosis.^1^ Despite its widespread use, the NAFLD framework has been criticized for its reliance on exclusion criteria, which may obscure the underlying metabolic pathogenesis and complicate both clinical diagnosis and research comparability.^2^ To address these shortcomings, the concept of metabolic dysfunction–associated fatty liver disease (MAFLD) was introduced in 2020, representing a paradigm shift from an exclusionary to an inclusionary definition.^2^ Metabolic dysfunction–associated fatty liver disease diagnosis requires evidence of steatosis together with overweight/obesity, T2DM, or specific metabolic abnormalities (e.g., increased waist circumference, hypertension, elevated triglycerides, reduced high-density lipoprotein cholesterol (HDL-C), prediabetes, insulin resistance, or elevated C-reactive protein level).^3^ Clinical studies in patients with type 2 diabetes have highlighted that MAFLD is frequently underdiagnosed in routine practice and is associated with a substantial risk of advanced fibrosis, emphasizing the need for improved identification and monitoring strategies.^4^ This reclassification underscores the central role of metabolic derangements in disease initiation and progression, facilitates risk stratification, and strengthens the integration of fatty liver disease into the broader spectrum of cardiometabolic disorders.^3^ Importantly, MAFLD highlights the systemic implications of fatty liver, positioning it not merely as a hepatic condition but as a manifestation of multisystem metabolic dysfunction.^5^ Building on this transition, hepatology societies in 2023 endorsed the nomenclature Metabolic dysfunction - associated steatotic liver disease (MASLD).^6^ MASLD retains the focus on metabolic drivers while removing stigmatizing terminology and simplifying diagnostic language.^7^ In addition, by avoiding rigid exclusion rules, MASLD provides a more flexible and clinically adaptable framework.^7^

Systemic inflammation plays a fundamental role in the initiation and progression of many chronic metabolic and hepatic disorders. In recent years, a variety of hematological and biochemical indices have been developed to quantify inflammatory activity and immune–nutritional balance using readily available clinical parameters. Among them, the systemic immune-inflammation index (SII) and the systemic inflammation response index (SIRI) integrate neutrophil, lymphocyte, monocyte, and platelet counts, providing a dynamic reflection of the interplay between innate and adaptive immunity.^8^^,^^9^ The prognostic nutritional index (PNI), derived from albumin concentration and lymphocyte counts, extends this framework by linking immune competence with nutritional status, which is particularly relevant in chronic liver disease.^10^ Likewise, the platelet-to-lymphocyte ratio (PLR) and lymphocyte-to-monocyte ratio (LMR) have been widely explored as indicators of systemic immune imbalance and fibrogenic activity. Beyond these established markers, several novel indices have been proposed to capture more complex inflammatory interactions.^11^^,^^12^ The aggregate index of systemic inflammation (AISI) incorporates multiple hematological variables into a single composite score, whereas the neutrophil-to-prealbumin ratio (NPAR) provides a sensitive marker of inflammation tightly coupled with protein metabolism.^13^ The neutrophil-to-lymphocyte ratio (NLR), one of the most extensively studied indices, has been consistently shown to increase in parallel with greater disease severity across diverse clinical settings.^12^ The inflammatory burden index (IBI), which combines C-reactive protein with leukocyte parameters, reflects both acute-phase and chronic inflammatory responses.^14^ More recently, the pan-immune-inflammation value (PIV), integrating neutrophils, lymphocytes, monocytes, and platelets, has emerged as a comprehensive measure of systemic immune activation.^15^ Together, these indices offer complementary insights into the inflammatory milieu of chronic diseases. Their application in the context of MASLD is particularly promising, as inflammation is a key link between metabolic dysregulation and hepatic fibrogenesis. Comparative assessment of these indices can clarify which markers correlate most consistently with disease severity and thereby guide targeted validation in prospective studies.

Previous studies have investigated associations between systemic inflammatory indices and MASLD, yet their potential link with hepatic fibrosis has not been adequately clarified.^16^ Considering the central role of inflammation in both steatosis and fibrogenesis, clarifying these links is of particular interest. The interactions between hepatic cells and microenvironments play a critical role in fibrosis progression, providing a mechanistic rationale for investigating systemic inflammatory indices in relation to liver stiffness and fibrogenesis.^17^ Vibration-controlled transient elastography (VCTE) offers a practical, noninvasive approach to capture hepatic changes: the controlled attenuation parameter (CAP) reflects steatosis through ultrasound attenuation, whereas liver stiffness measurement (LSM) quantifies fibrosis via shear wave velocity.^18^ In this study, prior work is extended by evaluating a broader spectrum of systemic inflammatory indices, incorporating both widely used markers and less frequently examined metrics. This expanded panel enables a more comprehensive characterization of inflammatory status and its potential relevance to MASLD and fibrosis. The objective is to investigate cross-sectional associations between these indices and hepatic outcomes, thereby contributing population-based evidence to refine understanding of the inflammatory component of metabolic liver disease.

## Materials and Methods

### Study Population

**Figure 1.**
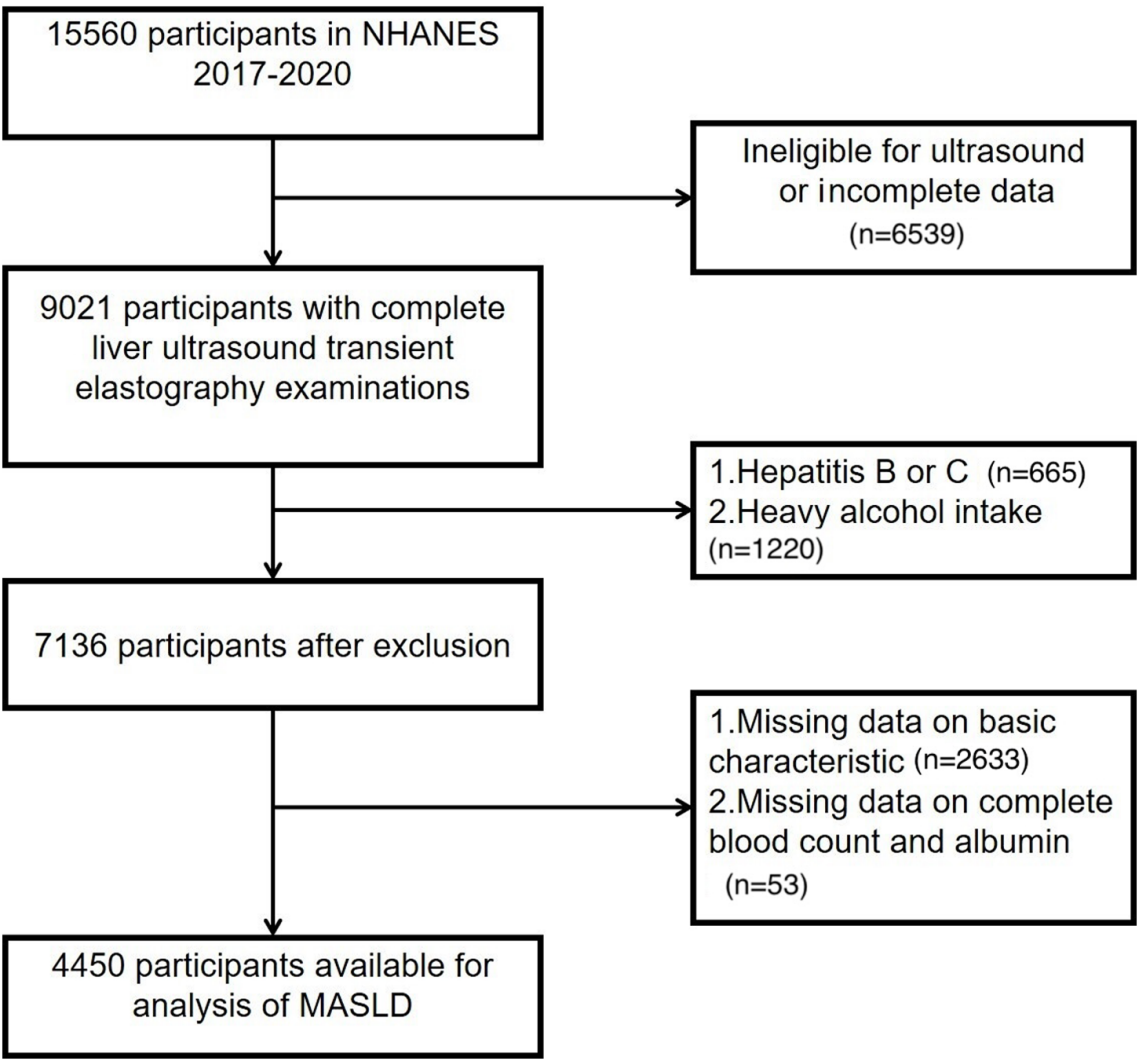
Flowchart of participant selection.

This study was based on de-identified, publicly available data from the 2017-2020 National Health and Nutrition Examination Survey (NHANES) cycle, which requires no additional ethical approval nor informed consent. Biochemical parameters, including fasting plasma glucose, lipid profile, and liver enzymes, were measured using standardized NHANES laboratory procedures. All assays were performed in NHANES-certified laboratories using automated analyzers according to manufacturer protocols. According to the institution’s guidelines for research involving human subjects, such analysis was deemed exempt from ethics approval and informed consent because the data contain no identifiable private information and the study participants have provided prior consent. The initial dataset comprised 15 560 participants. Exclusion criteria were applied sequentially to ensure data completeness and reduce potential confounding. Participants without liver elastography data, those with hepatitis B or C, and individuals reporting heavy alcohol consumption (≥3 drinks/day for men and ≥2 drinks/day for women) were excluded. Additional exclusions included participants with incomplete demographic or clinical characteristic data (n = 2633) and those with missing laboratory measures necessary for inflammatory indices calculation, such as complete blood count and albumin (n = 53). After applying these criteria, the final study group consisted of 4450 participants. A schematic representation of the cohort selection process is presented in Figure 1.

### Calculation of Systemic Inflammatory Indices

A comprehensive set of systemic inflammatory indices was derived from hematological and biochemical parameters available in the survey. Systemic immune-inflammation index was calculated as platelet × neutrophil / lymphocyte, and SIRI as neutrophil × monocyte / lymphocyte. Prognostic nutritional index was determined as albumin + 5 × lymphocyte, reflecting the combined effect of nutritional and immune status. Ratios such as PLR (platelet / lymphocyte), NLR (neutrophil / lymphocyte), and LMR (lymphocyte / monocyte) were computed to capture the relative balance of different circulating immune cells. More complex indices included AISI, defined as neutrophil × monocyte × platelet / lymphocyte, and PIV, expressed as platelet × neutrophil × monocyte / lymphocyte, both designed to integrate multiple immune components into a single composite score. In addition, NPAR was calculated as neutrophil / albumin, and IBI as C-reactive protein × neutrophil / lymphocyte, incorporating markers of systemic inflammation and protein metabolism.^8^^-^^15^ All indices were subsequently categorized into quartiles to evaluate their associations with MASLD and significant liver fibrosis (Supplementary Table 1).

### Definition of Metabolic Dysfunction–Associated Steatotic Liver Disease

Metabolic dysfunction–associated steatotic liver disease was defined by the presence of hepatic steatosis in individuals without significant alcohol consumption or viral hepatitis. Participants were considered to meet MASLD criteria if they exhibited hepatic fat accumulation alongside 1 or more of the following metabolic abnormalities: (1) overweight or central obesity, indicated by a body mass index (BMI) ≥ 25 kg/m^2^ or a waist circumference (WC) ≥ 94 cm in men and ≥80 cm in women; (2) impaired glycemic control, defined as fasting plasma glucose (FPG) ≥ 5.6 mmol/L (≥100 mg/dL), HbA1c ≥ 5.7%, a prior diagnosis of T2DM, or current antidiabetic therapy; (3) elevated blood pressure, specified as systolic/diastolic ≥ 130/85 mmHg or ongoing antihypertensive treatment; (4) hypertriglyceridemia, defined by triglyceride (TG) levels ≥ 1.70 mmol/L (≥ 150 mg/dL) or current use of lipid-lowering medications; and (5) reduced HDL-C, defined as <1.0 mmol/L (<40 mg/dL) for men or <1.3 mmol/L (<50 mg/dL) for women, or current lipid-lowering therapy. This definition emphasizes the central role of metabolic dysfunction in the development of hepatic steatosis and aligns with contemporary consensus recommendations for MASLD diagnosis.^6^

### Assessment of Liver Steatosis and Fibrosis

Hepatic steatosis and fibrosis were evaluated using VCTE. Participants were instructed to fast for a minimum of 3 hours prior to the examination. For each individual, at least 10 valid liver measurements were obtained, and quality control was ensured by maintaining an interquartile range to median ratio below 30%. Hepatic steatosis was defined by a CAP value of ≥260 dB/m,^19,20^ in accordance with previously validated thresholds. Significant liver fibrosis was assessed using LSM, with values ≥7 kPa considered indicative of significant fibrosis.^21^^,^^22^ This noninvasive approach allows reliable, quantitative assessment of both steatosis and fibrosis, facilitating standardized evaluation of hepatic pathology across large population-based cohorts. According to the recent guideline-recommended cutoffs (CAP >288 dB/m and LSM >8 kPa), a supplementary analysis was additionally performed using these thresholds.^23^

### Covariate Evaluation

Demographic and lifestyle information was collected using a standardized, self-administered questionnaire, encompassing age, sex, race/ethnicity, educational attainment, marital status, poverty-income ratio (PIR), alcohol intake, physical activity, and medication use. Poverty-income ratio was calculated as the ratio of family income to the federal poverty threshold, adjusted for family size and survey year, and categorized into 3 groups (<1.30, 1.30-3.50, >3.50) to indicate low, middle, and high socioeconomic status.^24^ Physical activity was quantified using the metabolic equivalent of task (MET) method, calculated as MET × weekly frequency × duration per session, with a value of 0 representing no activity. Participants were classified according to established recommendations, with a threshold of 600 MET-minutes per week indicating adequate activity for adults.^25^ Smoking exposure was assessed via serum cotinine concentrations.^26^^,^^27^ Diabetes mellitus was defined by fasting plasma glucose ≥7.0 mmol/L, HbA1c ≥ 6.5%, a self-reported physician diagnosis, or current use of antidiabetic medications.^28^ Hypertension was identified by systolic/diastolic blood pressure ≥130/80 mmHg or ongoing antihypertensive therapy.^29^ Alcohol consumption was classified into 2 categories: abstainers and moderate drinkers, defined as 1-2 drinks per day for men and 1 drink per day for women.^30^

### Statistical Analysis

Continuous variables are reported as means with SDs, and categorical variables as proportions. Weighted t-tests were applied to assess differences in continuous variables, while chi-square tests were used for categorical comparisons. The association between systemic inflammatory index and CAP, as well as LSM, was first examined using linear regression models. To account for potential confounding, 3 progressively adjusted models were constructed: the unadjusted model, a second model adjusting for demographic and socioeconomic factors (age, sex, ethnicity, education, marital status, PIR, physical activity, and BMI), and a fully adjusted model that additionally incorporated smoking, alcohol consumption, diabetes, and hypertension. Logistic regression analyses were subsequently conducted to evaluate the relationship of indices with MASLD and significant liver fibrosis, supplemented by subgroup analyses stratified by age, sex, and BMI. Restricted cubic spline analysis was used to explore potential nonlinear associations, and propensity score matching was performed to minimize selection bias before reassessing associations with logistic regression. Finally, the diagnostic accuracy of the individual index was evaluated using receiver operating characteristic (ROC) curve analysis, with area under the curve values compared to assess discriminative performance. To further assess the relationship between systemic inflammatory indices and fibrotic steatohepatitis, the FibroScan-AST (FAST) score was additionally calculated for participants with available LSM, CAP, and AST data, and analyzed as a noninvasive surrogate of at-risk metabolic dysfunction–associated steatohepatitis (MASH).^31^ All statistical analyses were conducted using R software version 4.1.0 (R Foundation for Statistical Computing; Vienna, Austria) and Stata version 16.0, (StataCorp LLC; College Station, TX, USA).

## Results

### Baseline Characteristics of the Study Population

A total of 4450 participants were included in the final analysis, and their baseline characteristics are summarized in Supplementary Table 2. Significant differences emerged between participants with MASLD and those without, particularly in demographic and lifestyle factors such as sex, ethnicity, marital status, smoking behavior, physical activity, diabetes, hypertension, and BMI (all *P* ≤ .05). Individuals with MASLD also exhibited higher values of age, WC, TC (total cholesterol), TG, HbA1c (glycated hemoglobin), FPG, SII, SIRI, PNI, NPAR, PIV, IBI, and AISI, whereas HDL-C and PLR were significantly lower (all *P* ≤ .05). When stratified by fibrosis status, participants with significant liver fibrosis differed significantly from those without in terms of sex, ethnicity, marital status, education level, physical activity, diabetes, hypertension, and BMI (all *P* ≤ .05). In contrast, TC and SIRI did not show significant difference between fibrosis groups. Detailed baseline characteristics are presented in Supplementary Table 2.

### Associations Between Systemic Inflammatory Indices with Controlled Attenuation Parameter and Liver Stiffness Measurement

The relationships between systemic inflammatory indices and CAP are summarized in Table 1. In Model 1, elevated SII, SIRI, LMR, IBI, and PNI were significantly and positively associated with CAP compared to the reference group, whereas PLR exhibited a negative association. These patterns persisted in both Model 2 and Model 3, indicating robustness to covariate adjustment. Specifically, SII, SIRI, LMR, IBI, and PNI showed positive correlations with CAP, with correlation coefficients ranging from 0.619 to 0.790 (all *P* < .05), while PLR demonstrated an inverse association (Q4 vs. Q1: β = −0.599, *P* = .027). The associations of NLR, NPAR, PIV, and AISI with CAP are detailed in Table 1. Table 2 presents the corresponding associations with LSM. In Model 1, higher SIRI, IBI, and AISI were significantly linked to increased LSM, while PLR remained inversely associated. Quantitatively, higher SIRI, PNI, and AISI were associated with increased LSM (Q4 vs. Q1: SIRI β = 0.619, *P* = .011; PNI β = 0.480, *P* = .035; AISI β = 0.480, *P* = .035), whereas PLR again showed a negative association (Q4 vs. Q1: β = −0.599, *P* = .027). These relationships were largely maintained in Models 2 and 3 after further adjustment. The associations of SII, LMR, NLR, NPAR, PIV, and PNI with LSM are reported in Table 2.

### Associations Between Systemic Inflammatory Indices for Metabolic Dysfunction–Associated Steatotic Liver Disease and Significant Liver Fibrosis

As summarized in Table 3, SII, SIRI, LMR, IBI, and PNI were significantly and positively associated with MASLD in the minimally adjusted model, whereas PLR showed an inverse relationship. Specifically, SII, SIRI, LMR, IBI, and PNI demonstrated positive associations with MASLD (SII OR 1.442, 95% CI 1.058-1.966, *P* = .021; SIRI OR 1.517, 95% CI 1.101-2.090, *P* = .011; LMR OR 1.423, 95% CI 1.047-1.934, *P* = .024; PNI OR 1.829, 95% CI 1.353-2.473, *P* < .001; IBI OR 1.788, 95% CI 1.296-2.466, *P* < .001), while PLR was inversely associated (OR 0.682, 95% CI 0.510-0.911, *P* = .009). These associations remained stable across Models 2 and 3. Findings for NLR, NPAR, PIV, and AISI are presented in Table 3. Table 4 presents the corresponding results for significant liver fibrosis. Higher SIRI, PNI, IBI, and AISI were associated with increased fibrosis risk (SIRI OR 1.423, 95% CI 1.047-1.935, *P* = .024; PNI OR 1.644, 95% CI 1.209-2.236, *P* = .002; AISI OR 1.445, 95% CI 1.052-1.986, *P* = .023; IBI OR 2.779, 95% CI 1.478-5.224, *P* = .002), whereas PLR demonstrated an inverse association (OR 0.666, 95% CI 0.490-0.906, *P* = .009). These patterns remained broadly consistent after further adjustment. Additional associations involving SII, LMR, NLR, NPAR, and PIV are presented in Table 4.

In addition, Supplementary Table 3 summarizes the associations between inflammatory indices and at-risk MASH. Systemic immune-inflammation index, SIRI, LMR, PNI, and IBI showed positive associations with MASH, whereas PLR remained inversely related. The data was additionally reanalyzed using CAP >288 dB/m and LSM >8 kPa. For MASLD (CAP >288 dB/m), SIRI, PNI, LMR, IBI, and SII remained positively associated across quartiles, whereas PLR continued to show an inverse association. Similarly, for significant liver fibrosis (LSM >8 kPa), higher SIRI, PNI, and IBI were associated with increased fibrosis risk, while PLR consistently demonstrated a protective pattern (Supplementary Table 4).

**Figure 2.**
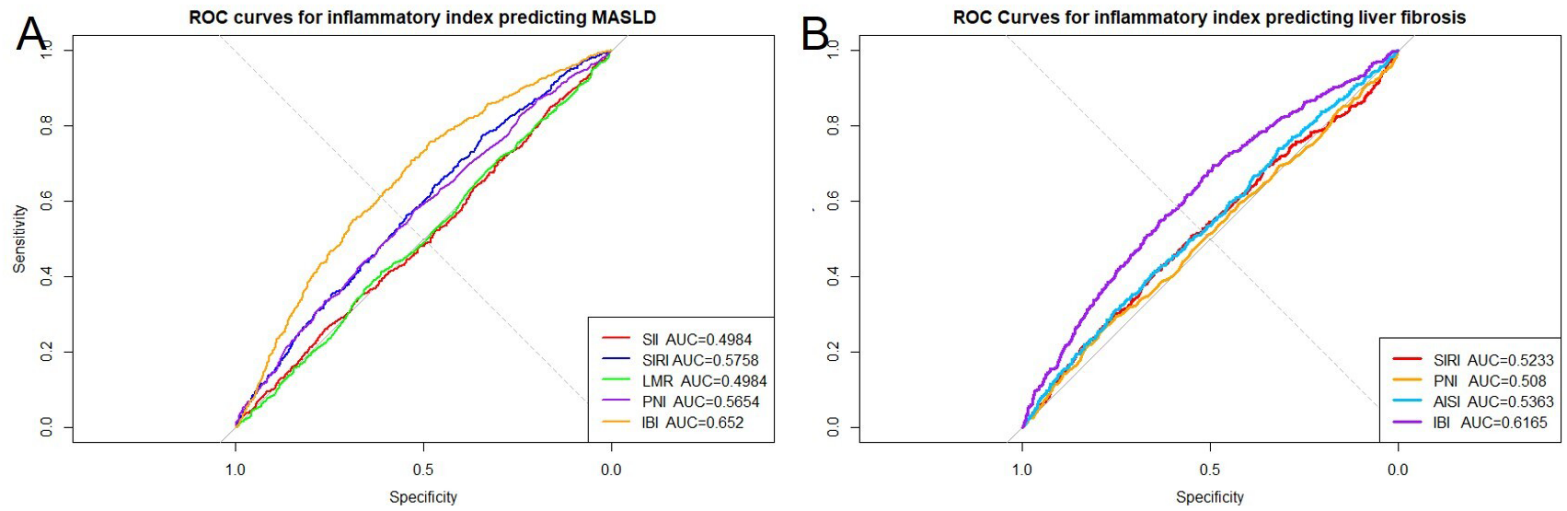
Receiver operating characteristic curves of systemic inflammatory indices for the diagnosis of (A) MASLD and (B) significant liver fibrosis.

**Figure 3.**
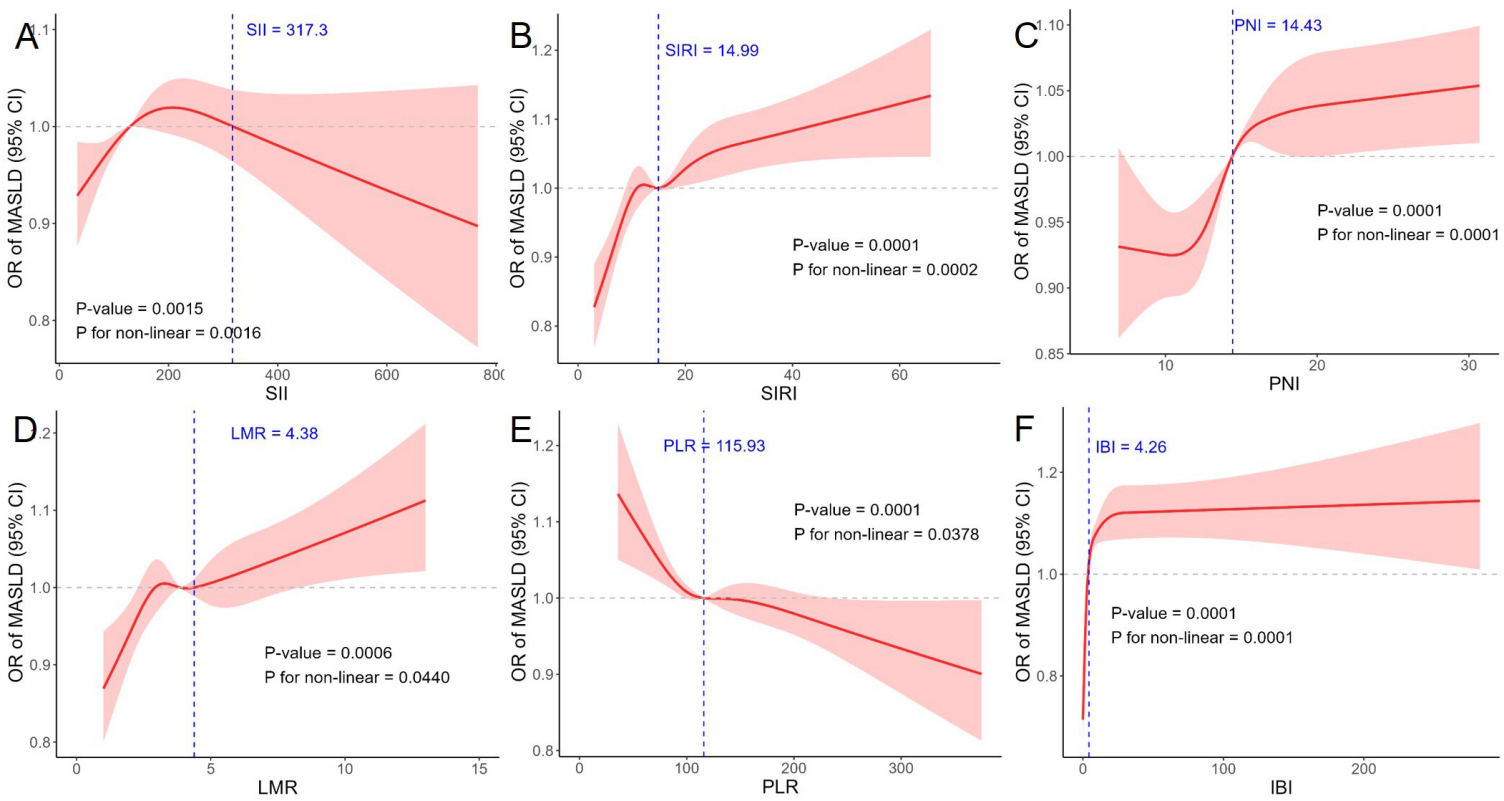
Restricted cubic spline analysis of the associations between MASLD and (A) SII, (B) SIRI, (C) PNI, (D) LMR, (E) PLR, and (F) IBI.

**Figure 4.**
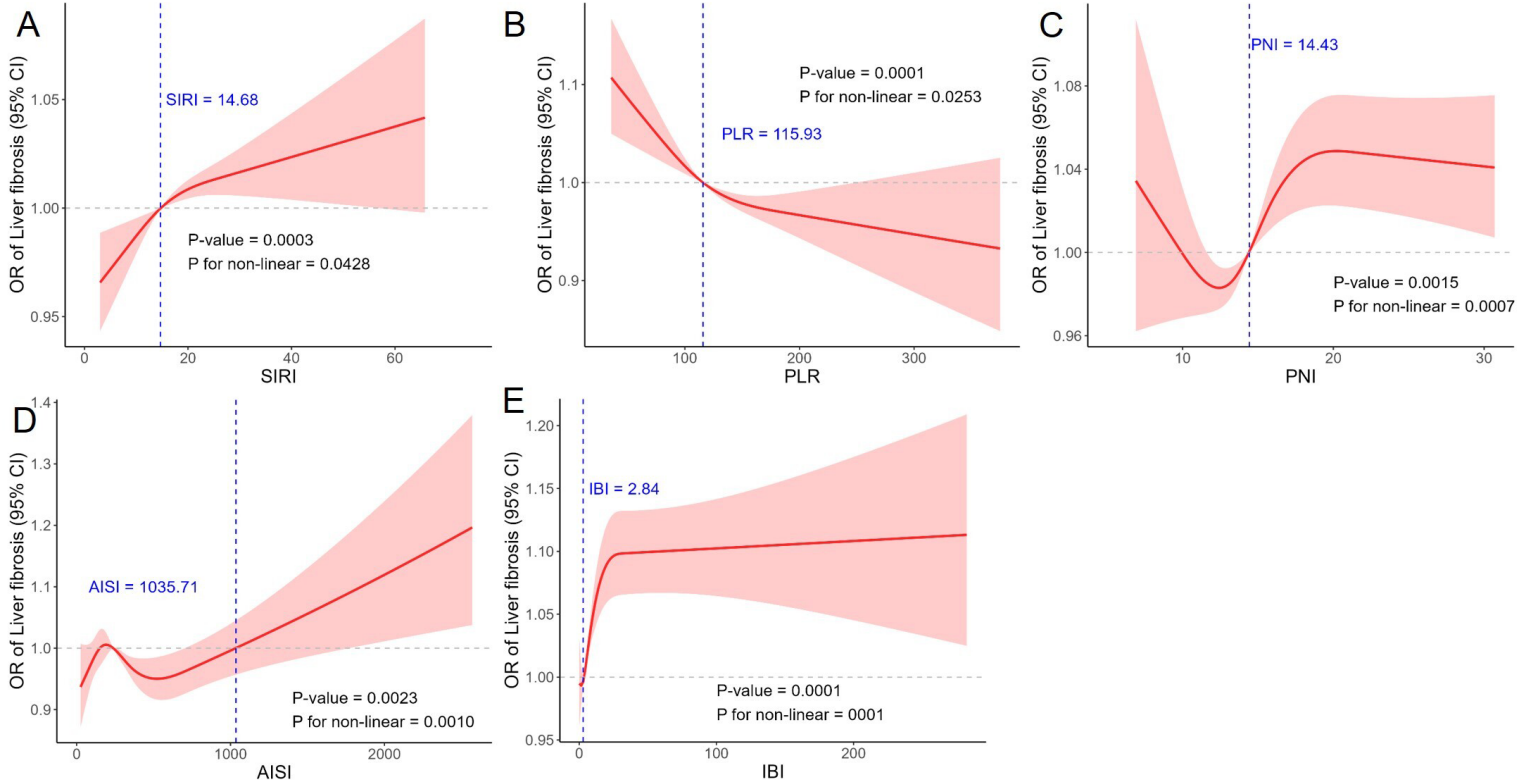
Restricted cubic spline analysis of the associations between significant liver fibrosis and (A) SIRI, (B) PLR, (C) PNI, (D) AISI, and (E) IBI.

Receiver operating characteristic curve analysis indicated generally low discriminative ability of the inflammatory indices. For MASLD, SIRI (AUROC [area under the receiver operating characteristic curve] = 0.576) and PNI (AUROC = 0.565) showed modest performance, while SII and LMR were close to non-informative. Inflammatory burden index reached an AUROC of 0.652, representing the highest among the tested indices (Figure 2; Supplementary Table 5). For significant liver fibrosis, SIRI, PNI, and AISI all produced AUROC values slightly above 0.5, reflecting limited diagnostic value, whereas IBI showed somewhat better discrimination with an AUROC of 0.604 (Supplementary Table 5). Restricted cubic spline analysis based on Model 3 further revealed nonlinear associations between several indices and both MASLD and fibrosis (Figures 3 and 4).

### Stratified and Sensitivity Analyses

Stratified multivariable regression analyses are illustrated in Supplementary Figures 1 and 2. A significant association was observed only between SIRI and significant liver fibrosis in the BMI-defined subgroup (*P* = .022), whereas no statistically significant associations emerged across other stratifications (all *P* > .05). Threshold effect analyses further showed no evidence of nonlinear relationships for any systemic inflammatory index with MASLD or significant fibrosis (all *P* for nonlinear >.05), indicating the absence of identifiable inflection points in these associations. To further assess robustness, propensity score matching followed by logistic regression was conducted for SIRI, PLR, PNI, IBI, and AISI in relation to significant liver fibrosis (refer to Supplementary Table 6). The results (Supplementary Tables 7 and 8) were consistent with the main analyses, supporting the stability of these findings. Given that the initial MASLD and non-MASLD groups were already well balanced (2133 vs. 2317), additional propensity score matching was not performed for MASLD.

## Discussion

Previous research from population-based cohorts linking systemic inflammatory markers with significant liver fibrosis assessed by transient elastography remains scarce.^16^^,^^32^ Although several markers have been studied, others have not been systematically investigated.^16^^,^^32^ Leveraging nationally representative NHANES data with VCTE assessments, a broad spectrum of inflammatory indicators were evaluated in relation to hepatic steatosis and fibrosis. The current study revealed that elevated SII, SIRI, LMR, and PNI were consistently associated with higher odds of MASLD, while PLR showed an inverse pattern. In the context of significant liver fibrosis, higher SIRI, PNI, and AISI were positively related, whereas PLR remained negatively associated. These findings remained robust after extensive adjustment for demographic and metabolic factors. Subgroup and propensity-matched analyses provided additional support, though significant signals emerged only for SIRI in the BMI-stratified fibrosis subgroup. Collectively, the evidence suggested that systemic inflammatory imbalance may contribute to susceptibility to MASLD and significant liver fibrosis. These results build upon but also extend previous population-based findings, offering a more comprehensive evaluation of multiple inflammatory indices simultaneously and providing a more comprehensive picture of how different hematological markers may parallel the spectrum of hepatic involvement.

Systemic inflammatory perturbations likely bridge metabolic dysfunction and hepatic injury through several convergent pathways.^33^ Adipose-tissue inflammation and lipid overflow stimulate the release of pro-inflammatory mediators such as tumor necrosis factor-alpha and interleukin-6 and promote chemokine-driven expansion of myelopoiesis with subsequent mobilization of neutrophils and monocytes to metabolic tissues and the liver.^34^^,^^35^ In parallel, gut dysbiosis and increased intestinal permeability permit translocation of microbial products (e.g., lipopolysaccharide) that engage hepatic pattern-recognition receptors on Kupffer cells and stellate cells, activating NF-κB–dependent signaling and sensitizing stellate cells to profibrogenic stimuli.^36^^,^^37^ Injured hepatocytes also release mitochondrial and other damage-associated molecular patterns that directly engage myeloid effectors and hepatic stellate cells, and cooperate with canonical profibrogenic mediators (notably transforming growth factor-β) and developmental programs to drive extracellular matrix accumulation.^38^^-^^40^ Furthermore, hypoxia and oxygen homeostasis disruption in metabolically stressed hepatic tissue can activate hypoxia-inducible factors, reprogramming cellular metabolism and upregulating fibrogenic genes, which enhance stellate cell activation and extracellular matrix deposition.^41^^,^^42^ Persistent low-grade inflammation may also induce immune checkpoint dysregulation and T cell exhaustion within the liver, impairing anti-fibrotic immune surveillance and promoting a pro-fibrogenic microenvironment.^43^ In population studies that use VCTE, hepatic steatosis and fibrosis are quantified respectively by CAP and LSM, and commonly used composite hematologic scores include the SII and the SIRI.^44^^,^^45^ These cellular and molecular cascades provide a plausible explanation for why peripheral composite indices reflecting balance of neutrophil, lymphocyte, monocyte, and platelet are associated with CAP (steatosis) and LSM (fibrosis) in population data, even if their individual discriminative performance is modest. Importantly, these mechanistic pathways help contextualize the clinical associations observed in this analysis, reinforcing the concept that systemic immune imbalance is an integral component of metabolic liver disease progression rather than a secondary epiphenomenon.

In the subgroup analyses, systemic inflammatory indices did not show significant associations with MASLD when stratified by age, sex, or BMI. This stands in contrast to prior reports that identified notable sex-specific differences.^16^ For example, Burnside et al demonstrated in a large Canadian cohort that the prevalence of MASLD was substantially higher in men than in women (46% vs. 24%), and that sex further modified the cardiometabolic risk profiles associated with MASLD.^46^ Such discrepancies may be attributed to differences in population characteristics, diagnostic strategies (e.g., CAP/LSM–based definition versus traditional fatty liver index), or the inflammatory markers examined. For significant liver fibrosis, the only notable subgroup effect observed in this study was a stronger association of SIRI among individuals with higher BMI, suggesting that adiposity may amplify the link between systemic inflammation and fibrotic burden. Collectively, these findings underscore the importance of considering demographic and metabolic factors as potential effect modifiers in future investigations and highlight the need for more standardized methodologies across cohorts to clarify population-specific vulnerabilities. Given these variations, further harmonized research across diverse populations will be crucial to determine whether the observed associations represent universal biological patterns or reflect context-dependent interactions.

To the authors’ knowledge, this is the first study to systematically evaluate the relationship between systemic inflammatory indices, MASLD, and significant liver fibrosis using VCTE data from a nationally representative cohort. Nonetheless, several limitations should be acknowledged. First, the cross-sectional nature of NHANES precludes causal inference, restricting the ability to determine temporal relationships between inflammatory activity and hepatic outcomes. Second, inflammatory responses are dynamic and may vary across different stages of MASLD progression; the present analysis could not capture these longitudinal changes. Third, the inflammatory indices assessed here are derived from standard hematological parameters rather than direct measures of inflammatory mediators, which may limit their biological specificity. Fourth, although NHANES provides detailed laboratory and imaging data, certain clinical variables—such as liver biopsy confirmation, longitudinal follow-up, or repeated biomarker assessments—were unavailable, potentially constraining the depth of inference. Future prospective and mechanistic studies are warranted to validate these findings and clarify how systemic inflammation contributes to MASLD and fibrosis progression.

In conclusion, in this large, nationally representative cross-sectional study, significant associations were observed between several systemic inflammatory indices and hepatic outcomes assessed by VCTE. Elevated levels of SII, SIRI, LMR, IBI, and PNI were positively related to MASLD, whereas PLR showed an inverse association. With respect to significant liver fibrosis, higher SIRI, PNI, IBI, and AISI were consistently associated with greater risk, while PLR again demonstrated a negative relationship. Although the discriminative performance of these indices was modest, the findings underscore a potential role of systemic inflammatory dysregulation in the pathogenesis of MASLD and fibrosis. Further longitudinal and mechanistic studies are needed to confirm these associations and clarify their clinical implications.

## Supplementary Materials

Supplementary Material

## Figures and Tables

**Table 1.** Correlations of Systemic Inflammatory Indices with Controlled Attenuation Parameter

Index	Model	Q1	Q2		Q3		Q4	
			OR (95% CI)	*P*	OR (95% CI)	*P*	OR (95% CI)	*P*
SII	model1	ref	1.647 (−9.126 to 5.831)	.666	4.465 (−3.202 to 12.132)	.254	9.939 (2.071-17.806)	.013
	model2	ref	1.851 (−5.505 to 9.207)	.622	5.264 (−1.976 to 12.505)	.154	6.682 (0.744-12.620)	.027
	model3	ref	1.829 (−4.202 to 7.859)	.552	5.247 (−1.926 to 12.420)	.152	8.148 (2.188-14.109)	.007
SIRI	model1	ref	13.228 (5.977-20.479)	<.001	16.165 (8.706-23.623)	<.001	24.187 (16.512-31.862)	<.001
	model2	ref	14.418 (7.496-21.341)	<.001	19.361 (12.264-26.457)	<.001	29.545 (21.991-37.098)	<.001
	model3	ref	8.653 (2.661-14.645)	.005	8.654 (2.473-14.835)	.006	13.997 (7.558-20.435)	<.001
PLR	model1	ref	−7.652 (−15.535 to 0.232)	.057	−10.888 (−18.743 to −3.032)	.007	−17.912 (−25.482 to −10.341)	<.001
	model2	ref	−5.644 (−12.965 to 1.677)	.131	−8.732 (−16.042 to −1.422)	.019	−16.834 (−23.866 to −9.803)	<.001
	model3	ref	−6.065 (−12.081 to −0.049)	.048	−7.218 (−12.838 to −1.598)	.012	−11.877 (−17.692 to −6.061)	<.001
LMR	model1	ref	1.624 (−5.665 to 8.913)	.662	17.508 (10.151-24.865)	<.001	19.586 (12.152-27.020)	<.001
	model2	ref	1.666 (−5.523 to 8.855)	.650	6.912 (0.862-12.963)	.025	8.166 (0.381-15.951)	.039
	model3	ref	3.725 (−2.312 to 9.763)	.226	8.128 (1.652-14.604)	.014	8.805 (2.283-15.327)	.008
PNI	model1	ref	7.122 (−0.353 to 14.596)	.062	12.706 (5.584-19.829)	<.001	25.245 (17.344-33.147)	<.001
	model2	ref	10.139 (2.792-17.486)	.007	16.902 (9.963-23.840)	<.001	29.795 (22.381-37.209)	<.001
	model3	ref	16.213 (10.039-22.388)	<.001	16.213 (10.039-22.388)	<.001	16.213 (10.039-22.388)	<.001
NLR	model1	ref	0.745 (−6.678 to 8.168)	.844	−4.481 (−12.090 to 3.127)	.248	1.755 (−5.056 to 8.566)	.613
	model2	ref	0.473 (−7.468 to 6.522)	.895	−6.770 (−13.887 to 0.347)	.062	−4.083 (−11.005 to 2.839)	.248
	model3	ref	0.171 (−5.485 to 5.828)	.953	−4.790 (−10.793 to 1.214)	.118	2.775 (−3.454 to 9.004)	.383
NPAR	model1	ref	10.907 (3.368-18.445)	.005	12.607 (5.317-19.897)	.001	15.640 (7.890-23.391)	<.001
	model2	ref	9.639 (2.502-16.776)	.008	10.292 (3.314-17.269)	.004	12.701 (5.267-20.135)	.001
	model3	ref	2.802 (−3.030 to 8.634)	.346	−1.578 (−7.731 to 4.575)	.615	−2.608 (−8.909 to 3.693)	.417
PIV	model1	ref	6.239 (−0.902 to 13.381)	.087	16.933 (9.460-24.406)	<.001	21.720 (14.461-28.980)	<.001
	model2	ref	3.303 (−3.585 to 10.190)	.347	12.778 (5.505-20.052)	<.001	13.820 (6.498-21.142)	<.001
	model3	ref	0.188 (−6.066 to 5.690)	.951	0.910 (−5.391 to 7.211)	.777	0.595 (−5.677 to 6.867)	.852
IBI	model1	ref	15.960 (9.072-22.849)	−1.852	39.609 (32.794-46.424)	<.001	48.995 (41.490-56.499)	<.001
	model2	ref	4.115 (−1.642 to 9.873)	.161	16.213 (10.039-22.388)	<.001	23.039 (16.442-29.635)	<.001
	model3	ref	4.230 (−1.498 to 9.958)	.148	14.825 (8.793-20.858)	<.001	18.146 (11.717-24.575)	<.001
AISI	model1	ref	0.614 (−7.647 to 6.419)	.864	−3.157 (−10.893 to 4.579)	.424	−1.647 (−9.126 to 5.831)	.666
	model2	ref	−5.427 (−12.985 to 2.130)	.878	1.889 (−9.593 to 5.816)	.631	1.945 (−9.751 to 5.860)	.625
	model3	ref	−3.501 (−9.454 to 2.452)	.249	2.223 (−3.595 to 8.041)	.454	1.708 (−4.312 to 7.729)	.578

AISI, aggregate index of systemic inflammation; IBI, inflammatory burden index; LMR, lymphocyte-to-monocyte ratio; NLR, neutrophil-to-lymphocyte ratio; NPAR, neutrophil percentage to albumin ratio; OR, odds ratio; PIV, pan-immune-inflammation value; PLR, platelet-to-lymphocyte ratio; PNI, prognostic nutritional index; SIRI, systemic inflammation response index; SII, systemic immune-inflammation index.

**Table 2.** Correlations of Systemic Inflammatory Indices with Liver Stiffness Measurement

Index	Model	Q1	Q2		Q3		Q4	
			OR (95% CI)	*P*	OR (95% CI)	*P*	OR (95% CI)	*P*
SII	model1	ref	0.022 (−0.495 to 0.452)	.929	0.132 (−0.334 to 0.599)	.578	0.203 (−0.240 to 0.646)	.369
	model2	ref	0.180 (−0.335 to 0.694)	.493	0.007 (−0.506 to 0.491)	.977	0.057 (−0.454 to 0.341)	.781
	model3	ref	0.217 (−0.272 to 0.707)	.384	0.090 (−0.379 to 0.558)	.707	0.015 (−0.360 to 0.390)	.937
SIRI	model1	ref	0.025 (−0.521 to 0.471)	.921	−0.296 (−0.774 to 0.182)	.224	0.685 (0.237-1.133)	.003
	model2	ref	0.002 (−0.452 to 0.456)	.993	0.265 (−0.183 to 0.713)	.246	1.680 (1.222-2.310)	.001
	model3	ref	0.003 (−0.453 to 0.458)	.991	0.145 (−0.593 to 0.303)	.526	0.619 (0.145-1.093)	.011
PLR	model1	ref	−0.434 (−0.946 to 0.078)	.096	−0.438 (−0.941 to 0.065)	.088	−0.639 (−1.133 to 0.144)	.011
	model2	ref	−0.348 (−0.840 to 0.144)	.166	−0.488 (−1.008 to 0.032)	.066	−0.547 (−1.016 to 0.078)	.022
	model3	ref	−0.337 (−0.821 to 0.147)	.173	−0.383 (−0.844 to 0.077)	.103	−0.599 (−1.129 to −0.069)	.027
LMR	model1	ref	0.086 (−0.632 to 0.460)	.757	0.535 (−0.952 to 0.118)	.452	0.415 (−0.856 to 0.027)	.066
	model2	ref	0.194 (−0.359 to 0.747)	.491	−0.171 (−0.574 to 0.231)	.404	0.041 (−0.487 to 0.406)	.858
	model3	ref	0.268 (−0.256 to 0.792)	.316	−0.111 (−0.501 to 0.280)	.663	0.020 (−0.443 to 0.404)	.927
PNI	model1	ref	0.109 (−0.296 to 0.513)	.223	0.213 (−0.251 to 0.677)	.369	0.582 (0.100-1.064)	.018
	model2	ref	0.151 (−0.239 to 0.541)	.447	0.329 (−0.125 to 0.784)	.156	0.385 (−0.183 to 0.713)	.246
	model3	ref	0.041 (−0.352 to 0.434)	.839	0.176 (−0.274 to 0.626)	.443	0.424 (−0.021 to 0.868)	.062
NLR	model1	ref	0.278 (−0.175 to 0.731)	.229	0.056 (−0.332 to 0.444)	.778	0.116 (−0.336 to 0.568)	.615
	model2	ref	0.224 (−0.216 to 0.665)	.318	0.003 (−0.384 to 0.391)	.986	0.082 (−0.379 to 0.543)	.728
	model3	ref	0.237 (−0.189 to 0.663)	.276	0.060 (−0.301 to 0.420)	.745	0.266 (−0.189 to 0.721)	.252
NPAR	model1	ref	0.371 (−0.107 to 0.850)	.128	0.370 (0.009-0.731)	.044	0.611 (0.237-0.985)	.001
	model2	ref	0.418 (−0.044 to 0.880)	.076	0.354 (−0.002 to 0.710)	.052	0.561 (0.176-0.946)	.004
	model3	ref	0.266(−0.170 to 0.702)	.232	0.091 (−0.254 to 0.437)	.605	0.174 (−0.182 to 0.530)	.338
PIV	model1	ref	0.068 (−0.298 to 0.434)	.716	−0.029 (−0.440 to 0.382)	.89	0.601 (0.232-0.969)	.001
	model2	ref	−0.056 (−0.410 to 0.298)	.758	0.062 (−0.402 to 0.525)	.794	0.239 (−0.149 to 0.627)	.228
	model3	ref	−0.156 (−0.495 to 0.182)	.365	0.200 (−0.244 to 0.645)	.377	−0.108 (−0.460 to 0.244)	.548
IBI	model1	ref	0.182 (−0.233 to 0.597)	.389	0.333 (−0.153 to 0.819)	.179	0.268 (−0.087 to 0.622)	.139
	model2	ref	0.233 (−0.167 to 0.632)	.254	0.356 (−0.125 to 0.838)	.147	0.228 (−0.132 to 0.588)	.214
	model3	ref	0.188 (−0.207 to 0.582)	.352	0.218 (−0.251 to 0.688)	.362	0.039 (−0.382 to 0.304)	.822
AISI	model1	ref	0.203 (−0.165 to 0.571)	.279	0.447 (−0.036 to 0.931)	.07	0.633 (0.261-1.006)	.001
	model2	ref	0.111 (−0.247 to 0.468)	.544	0.369 (−0.090 to 0.828)	.115	0.446 (0.072-0.820)	.019
	model3	ref	0.045 (−0.297 to 0.388)	.795	0.103 (−0.237 to 0.442)	.553	0.480 (0.034-0.926)	.035

AISI, aggregate index of systemic inflammation; IBI, inflammatory burden index; LMR, lymphocyte-to-monocyte ratio; NLR, neutrophil-to-lymphocyte ratio; NPAR, neutrophil percentage to albumin ratio; OR, odds ratio; PIV, pan-immune-inflammation value; PLR, platelet-to-lymphocyte ratio; PNI, prognostic nutritional index; SIRI, systemic inflammation response index; SII, systemic immune-inflammation index.

**Table 3.** Correlations of Systemic Inflammatory Indices with Metabolic Dysfunction–Associated Steatotic Liver Disease

Index	Model	Q1	Q2		Q3		Q4	
			OR (95% CI)	*P*	OR (95% CI)	*P*	OR (95% CI)	*P*
SII	model1	ref	0.937 (0.732-1.199)	.604	0.920 (0.714-1.185)	.518	1.457 (1.125-1.888)	.004
	model2	ref	1.101 (0.855-1.417)	.455	1.268 (0.966-1.664)	.087	1.386 (1.070-1.796)	.013
	model3	ref	1.125 (0.845-1.498)	.419	1.437 (1.061-1.946)	.019	1.442 (1.058-1.966)	.021
SIRI	model1	ref	1.318 (1.021-1.700)	.034	1.327 (1.029-1.711)	.029	1.837 (1.421-2.375)	<.001
	model2	ref	1.028 (0.767-1.378)	.852	1.483 (1.143-1.925)	.003	2.214 (1.683-2.282)	<.001
	model3	ref	1.075 (0.798-1.448)	.633	1.230 (0.916-1.653)	.168	1.517 (1.101-2.090)	.011
PLR	model1	ref	0.829 (0.639-1.076)	.159	0.712 (0.551-0.921)	.01	0.688 (0.533-0.889)	.004
	model2	ref	0.880 (0.681-1.137)	.007	0.737 (0.570-0.952)	.02	0.682 (0.528-0.882)	.004
	model3	ref	0.796 (0.588-1.076)	.138	0.716 (0.532-0.962)	.027	0.682 (0.510-0.911)	.009
LMR	model1	ref	0.980 (0.759-1.266)	.878	0.890 (0.695-1.139)	.354	1.331 (1.035-1.713)	.026
	model2	ref	1.099 (0.854-1.415)	.462	1.260 (0.968-1.640)	.086	1.399 (1.076-1.818)	.012
	model3	ref	1.214 (0.914-1.613)	.181	1.396 (1.015-1.919)	.041	1.423 (1.047-1.934)	.024
PNI	model1	ref	1.437 (1.120-1.843)	.004	1.437 (1.120-1.843)	.004	1.813 (1.403-2.342)	<.001
	model2	ref	1.265 (0.944-1.695)	.007	1.498 (1.129-1.989)	.005	1.845 (1.385-2.457)	<.001
	model3	ref	1.303 (0.966-1.759)	.083	1.525 (1.135-2.049)	.005	1.829 (1.353-2.473)	<.001
NLR	model1	ref	1.038 (0.807-1.336)	.77	0.945 (0.736-1.214)	.659	1.106 (0.928-1.318)	.259
	model2	ref	1.011 (0.783-1.305)	.932	0.879 (0.684-1.129)	.312	1.064 (0.794-1.426)	.677
	model3	ref	1.045 (0.776-1.408)	.771	0.922 (0.685-1.242)	.593	0.836 (0.618-1.131)	.246
NPAR	model1	ref	0.920 (0.714-1.185)	.518	1.459 (1.125-1.894)	.004	1.535 (1.190-1.981)	.001
	model2	ref	0.944 (0.719-1.240)	.679	1.407 (1.086-1.823)	.01	1.358 (1.047-1.762)	.021
	model3	ref	1.159 (0.864-1.555)	.326	0.911 (0.665-1.248)	.562	0.816 (0.600-1.110)	.195
PIV	model1	ref	1.086 (0.837-1.409)	.534	1.507 (1.163-1.953)	.002	1.666 (1.293-2.145)	<.001
	model2	ref	1.019 (0.782-1.329)	.887	1.391 (1.064-1.820)	.016	1.386 (1.060-1.812)	.017
	model3	ref	0.886 (0.650-1.209)	.447	0.937 (0.685-1.281)	.682	0.908 (0.662-1.245)	.548
IBI	model1	ref	1.651 (1.269-2.149)	<.001	3.260 (2.509-4.237)	<.001	4.091 (3.119-5.366)	<.001
	model2	ref	1.178 (0.886-1.565)	.26	1.734 (1.286-2.338)	<.001	2.084 (1.540-2.821)	<.001
	model3	ref	1.177 (0.874-1.584)	.284	1.667 (1.229-2.262)	.001	1.788 (1.296-2.466)	<.001
AISI	model1	ref	0.972 (0.749-1.261)	.832	1.552 (1.201-2.005)	.001	1.499 (1.165-1.928)	.002
	model2	ref	0.925 (0.711-1.203)	.56	1.496 (1.159-1.932)	.002	1.375 (1.062-1.779)	.016
	model3	ref	1.000 (0.751-1.331)	.997	0.799 (0.589-1.084)	.152	0.873 (0.649-1.175)	.371

AISI, aggregate index of systemic inflammation; IBI, inflammatory burden index; LMR, lymphocyte-to-monocyte ratio; NLR, neutrophil-to-lymphocyte ratio; NPAR, neutrophil percentage to albumin ratio; OR, odds ratio; PIV, pan-immune-inflammation value; PLR, platelet-to-lymphocyte ratio; PNI, prognostic nutritional index; SIRI, systemic inflammation response index; SII, systemic immune-inflammation index.

**Table 4.** Correlations of Systemic Inflammatory Indices with Liver Fibrosis

Index	Model	Q1	Q2		Q3		Q4	
			OR (95% CI)	*P*	OR (95% CI)	*P*	OR (95% CI)	*P*
SII	model1	ref	0.996 (0.751-1.321)	.977	0.890 (0.668-1.187)	.428	0.896 (0.661-1.214)	.477
	model2	ref	1.120 (0.845-1.485)	.431	1.053 (0.783-1.417)	.732	1.089 (0.790-1.500)	.604
	model3	ref	1.194 (0.884-1.613)	.247	1.220 (0.892-1.669)	.214	1.215 (0.863-1.710)	.264
SIRI	model1	ref	1.041 (0.768-1.411)	.796	1.275 (0.949-1.713)	.106	1.434 (1.063-1.933)	.018
	model2	ref	1.085 (0.796-1.477)	.607	1.210 (0.873-1.678)	.252	1.680 (1.222-2.310)	.001
	model3	ref	0.954 (0.679-1.341)	.205	1.156 (0.834-1.604)	.384	1.423 (1.047-1.935)	.024
PLR	model1	ref	0.697 (0.520-0.935)	.016	0.710 (0.530-0.952)	.022	0.592 (0.441-0.795)	<.001
	model2	ref	0.713 (0.532-0.954)	.023	0.743 (0.553-0.998)	.048	0.595 (0.441-0.803)	.001
	model3	ref	0.771 (0.573-1.039)	.087	0.710 (0.526-0.960)	.026	0.666 (0.490-0.906)	.009
LMR	model1	ref	0.889 (0.671-1.177)	.41	0.819 (0.611-1.099)	.184	0.945 (0.702-1.273)	.71
	model2	ref	1.043 (0.782-1.390)	.774	1.014 (0.747-1.377)	.929	1.191 (0.859-1.650)	.295
	model3	ref	1.153 (0.847-1.569)	.366	1.072 (0.783-1.469)	.663	1.266 (0.901-1.778)	.174
PNI	model1	ref	1.204 (0.893-1.623)	.223	1.138 (0.844-1.534)	.397	1.749 (1.300-2.352)	<.001
	model2	ref	1.284 (0.951-1.734)	.103	1.264 (0.934-1.711)	.132	1.954 (1.448-2.636)	<.001
	model3	ref	1.220 (0.877-1.696)	.238	1.135 (0.822-1.568)	.442	1.644 (1.209-2.236)	.002
NLR	model1	ref	1.101 (0.831-1.460)	.502	0.991 (0.737-1.333)	.954	0.894 (0.669-1.195)	.45
	model2	ref	1.060 (0.793-1.417)	.695	0.961 (0.712-1.298)	.796	0.851 (0.631-1.147)	.292
	model3	ref	1.080 (0.807-1.444)	.606	0.999 (0.739-1.350)	.996	0.988 (0.722-1.351)	.939
NPAR	model1	ref	1.132 (0.826-1.550)	.44	1.287 (0.942-1.759)	.112	1.322 (0.978-1.787)	.072
	model2	ref	1.154 (0.842-1.583)	.373	1.256 (0.919-1.716)	.153	1.262 (0.927-1.719)	.14
	model3	ref	1.021 (0.742-1.406)	.898	0.986 (0.710-1.369)	.933	0.886 (0.643-1.220)	.458
PIV	model1	ref	0.920 (0.668-1.267)	.609	0.893 (0.643-1.241)	.501	1.416 (1.046-1.919)	.025
	model2	ref	0.856 (0.617-1.189)	.355	1.181 (0.849-1.643)	.324	1.364 (0.984-1.891)	.063
	model3	ref	0.772 (0.551-1.082)	.133	1.059 (0.747-1.500)	.748	0.842 (0.593-1.197)	.338
IBI	model1	ref	2.206 (1.059-4.595)	.035	4.700 (2.504-8.822)	<.001	7.350 (4.027-13.414)	<.001
	model2	ref	1.575 (0.753-3.295)	.227	2.344 (1.211-4.536)	.011	3.483 (1.874-6.475)	<.001
	model3	ref	1.512 (0.745-3.069)	.252	2.112 (1.069-4.173)	.031	2.779 (1.478-5.224)	.002
AISI	model1	ref	1.087 (0.791-1.494)	.606	1.221 (0.893-1.670)	.211	1.608 (1.184-2.183)	.002
	model2	ref	1.046 (0.757-1.445)	.785	1.182 (0.861-1.623)	.301	1.486 (1.078-2.049)	.015
	model3	ref	0.975 (0.692-1.374)	.887	1.089 (0.774-1.532)	.626	1.445 (1.052-1.986)	.023

AISI, aggregate index of systemic inflammation; IBI, inflammatory burden index; LMR, lymphocyte-to-monocyte ratio; NLR, neutrophil-to-lymphocyte ratio; NPAR, neutrophil percentage to albumin ratio; OR, odds ratio; PIV, pan-immune-inflammation value; PLR, platelet-to-lymphocyte ratio; PNI, prognostic nutritional index; SIRI, systemic inflammation response index; SII, systemic immune-inflammation index.

## Data Availability

The data that support the findings of this study are available on request from the corresponding author.
