# Supplementary material for: Association of Systemic Inflammatory Indices with Metabolic Dysfunction–Associated Steatotic Liver Disease and Liver Fibrosis
Source: Turk J Gastroenterol. 2026 Feb 12;37(4):497–509. doi: 10.5152/tjg.2026.25656 (PMC13047331; doi:10.5152/tjg.2026.25656)
Supplement: Supplementary Material [file supplementary_material.pdf]

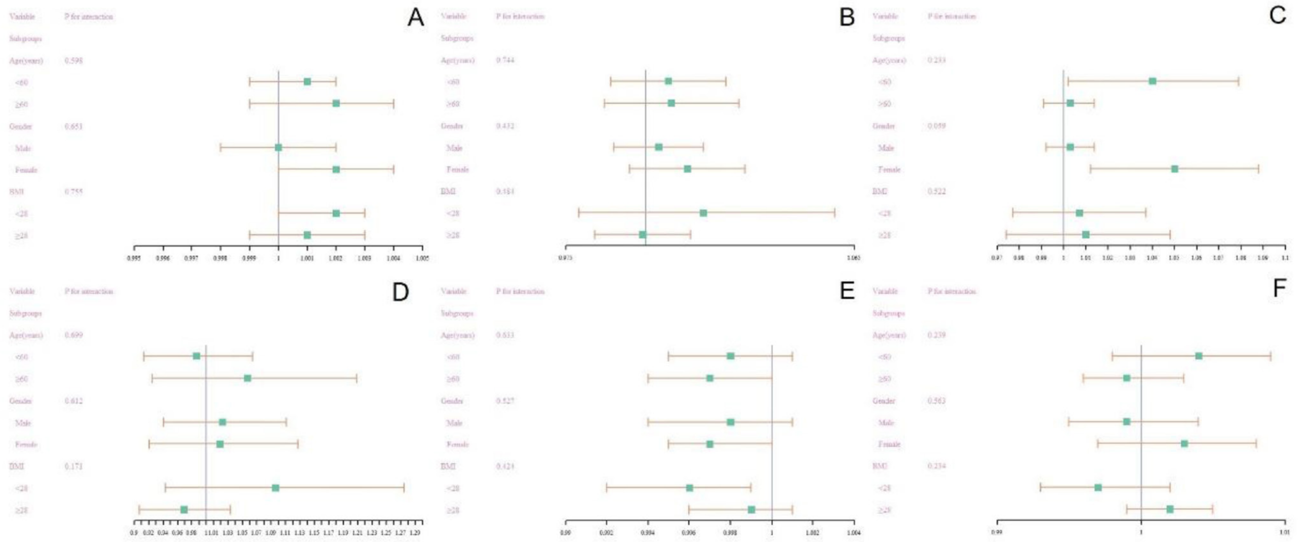

Supplementary Figure 1. Subgroup analyses of the associations between MASLD and (A) SII, (B) SIRI, (C) PNI, (D) LMR, (E) PLR, and (F) IBI.

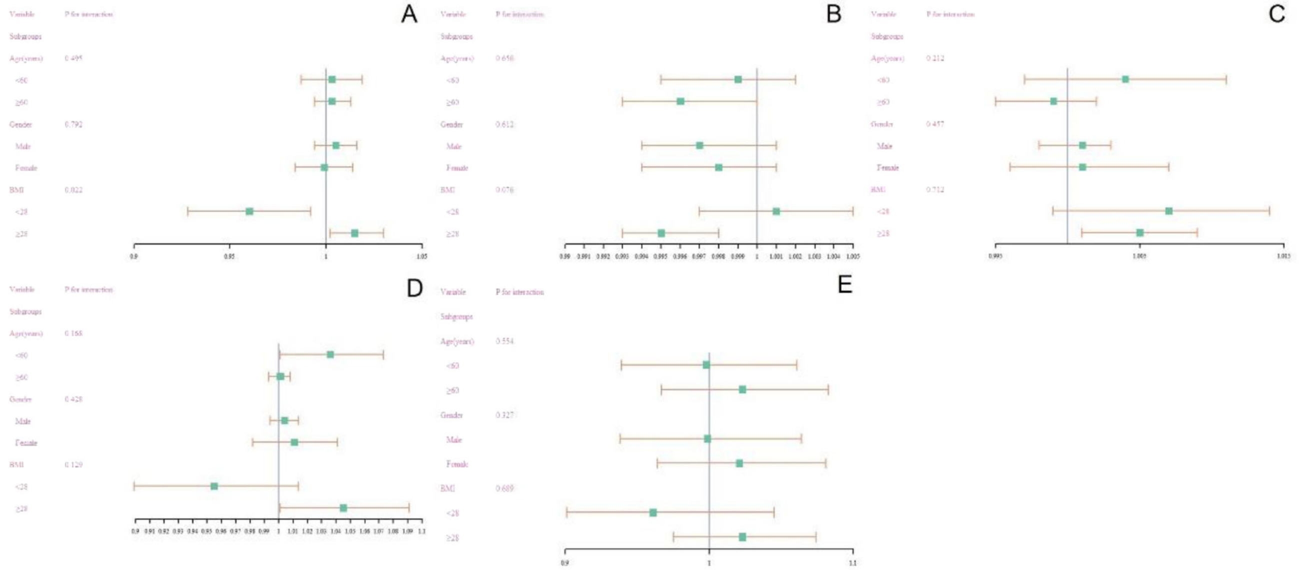

Supplementary Figure 2. Subgroup analyses of the associations between liver fibrosis and (A) SIRI, (B) PNI, (C) AISI, (D) PLR, and (E) IBI.

Supplementary Table 1. Interquartile grouping criteria for systemic inflammatory indices

Index	Quartiles 1	Quartiles 2	Quartiles 3	Quartiles 4
SII	<90.7856	90.7856-129.6256	129.6256-181.6552	≥181.6552
SIRI	<1.321429	1.321429-1.870968	1.870968-2.666667	≥2.666667
PLR	<10.69286	10.69286-14.73333	14.73333-20.125	≥20.125
LMR	<91.86508	91.86508-115.8359	115.8359-146.0833	≥146.0833
PNI	<20.3925	20.3925-38.5350	38.535-63.0925	≥63.0925
NLR	<3	3-3.8571	3.8571-5	≥5
NPAR	<12.3251	12.3251-14.0714	14.0714-15.85	≥15.85
PIV	<151.3979	151.3979-237.5243	237.5243-370.8418	≥370.8418
IBI	<0.6608	0.6608-1.0075	1.0075-1.5143	≥1.5143
AISI	<1.4434	1.4434-3.5227	3.5227-8.7062	≥8.7062

SII = Systemic immune-inflammation index, SIRI = Systemic inflammation response index, PLR = Platelet-to-lymphocyte ratio, LMR = Lymphocyte-to-monocyte ratio, PNI = Prognostic nutritional index, NLR = Neutrophil-to-lymphocyte ratio, NPAR = Neutrophil percentage to albumin ratio, PIV = Pan-immune-inflammation value, IBI = Inflammatory burden index, AISI = Aggregate index of systemic inflammation.

Supplementary Table 2. Baseline characteristics of participants

	Non-MASLD (n=2133)	MASLD (n=2317)	*P*-value	Non- Significant liver fibrosis (n=3380)	Significant liver fibrosis (n=1070)	*P*-value
Age (year)	44.51(0.80)	52.53(0.69)	<0.001	47.54(0.60)	52.16(1.18)	<0.001
Gender(n,%)			<0.001			<0.001
Male	923(43.27)	1181(50.97)		1508(44.62)	596(55.70)	
Female	1210(56.73)	1433(49.03)		1872(55.38)	474(44.30)	
Race(n,%)			<0.001			0.007
Mexican American	165(7.74)	311(13.42)		354(10.47)	122(11.40)	
Other Hispanic	204(9.56)	233(10.06)		328(9.70)	109(10.19)	
Non-Hispanic white	784(36.76)	912(39.36)		1290(38.17)	406(37.94)	
Non-Hispanic black	614(28.79)	494(21.32)		815(24.11)	293(27.38)	
Other	366(17.15)	367(15.84)		593(17.54)	140(13.08)	
Education level(n,%)			0.312			0.002
Less than high school	298(13.97)	406(17.52)		507(15.00)	197(18.41)	
High school or equivalent	477(22.36)	523(22.57)		740(21.89)	260(24.30)	
Above high school	1358(63.67)	1388(59.91)		2133(63.11)	613(57.29)	
Marital status (n, %)			<0.001			0.004
Married/cohabitant	1205(56.49)	1482(63.96)		2284(60.68)	412(58.69)	
Widowed/divorced/separated	458(21.47)	523(22.57)		800(21.25)	184(26.21)	
Never married	470(22.03)	312(13.47)		680(18.07)	106(15.10)	
Poverty income ratio (n, %)			0.329			0.081
<1.30	557(26.11)	574(24.77)		2050(60.65)	637(59.54)	
1.30-3.50	795(37.27)	912(39.36)		711(21.04)	270(25.23)	
>3.50	781(36.62)	831(35.87)		619(18.31)	163(15.23)	
Drinking status (n, %)			0.065			0.102
Non	745(34.93)	871(37.59)		1205(35.65)	411(38.41)	
Low to moderate	1388(65.07)	1446(62.41)		2175(64.35)	659(61.59)	
Smoking habits (n, %)			<0.001			0.856
Never	777(36.43)	919 (39.66)		1294(38.28)	402 (37.57)	
Moderate	845(39.62)	986(42.56)		1383(40.92)	448(41.87)	
Heavy	511(23.95)	412(17.78)		703(20.80)	220(20.56)	

(*Continued*)

Supplementary Table 2. Baseline characteristics of participants (*Continued*)

	Non-MASLD (n=2133)	MASLD (n=2317)	*P*-value	Non- Significant liver fibrosis (n=3380)	Significant liver fibrosis (n=1070)	*P*-value
Physical activity (n, %)			<0.001			0.023
Never	453(21.24)	644(27.79)		800(23.67)	297(27.76)	
Insufficient	242(11.35)	291(12.56)		406(12.01)	127(11.87)	
Constant	1438(67.42)	1382(59.65)		2174(64.32)	646(60.37)	
Diabetes(n, %)			<0.001			<0.001
Yes	216(10.13)	741(31.98)		551(16.30)	406(37.94)	
No	1917(89.87)	1576(68.02)		2829(83.70)	664(62.06)	
Hypertension(n, %)			<0.001			<0.001
Yes	969(45.43)	1571(67.80)		1787(52.87)	753(70.37)	
No	1164(54.57)	746(32.20)		1593(47.13)	317(29.63)	
BMI (kg/m2)			<0.001			<0.001
<28	1435(67.28)	1753(75.66)		1727(51.09)	272(25.42)	
≥28	698(32.72)	564(24.34)		1653(48.91)	798(74.58)	
WC(cm)	91.00(0.57)	108.70(0.62)	<0.001	97.11(0.53)	110.09(0.97)	<0.001
TG (mmol/L)	0.91(0.02)	1.48(0.03)	<0.001	1.15(0.02)	1.37(0.05)	<0.001
TC (mmol/L)	4.73(0.05)	4.93(0.05)	<0.001	4.88(0.04)	4.66(0.08)	0.256
LDL (mmol/L)	2.80(0.05)	2.97(0.04)	0.135	2.93(0.04)	2.73(0.06)	0.647
HDL (mmol/L)	1.51(0.02)	1.29(0.02)	<0.001	1.42(0.01)	1.30(0.03)	0.897
FPG(mmol/L)	5.63(0.03)	6.53(0.08)	<0.001	5.91(0.06)	6.69(0.13)	<0.001
Hb1Ac(%)	5.44(0.03)	5.90(0.04)	<0.001	5.57(0.02)	6.05(0.07)	<0.001
SII	136.75(2.90)	141.44(2.37)	<0.001	132.25(2.60)	140.93(5.67)	0.013
SIRI	13.78(0.28)	15.64(0.34)	0.011	14.52(0.26)	15.39(0.43)	0.213
PLR	134.64(2.11)	126.82(1.99)	<0.001	132.50(1.68)	124.11(2.05)	0.021
LMR	3.94(0.07)	3.92(0.07)	0.978	3.94(0.05)	3.98(0.11)	0.858
PNI	13.72(0.16)	14.43(0.15)	<0.001	14.00(0.13)	14.34(0.24)	0.001
NLR	2.36(0.06)	2.26(0.06)	0.279	2.31(0.05)	2.33(0.08)	0.475
NPAR	13.86(0.13)	14.43(0.11)	0.001	14.04(0.10)	14.53(0.20)	0.001
PIV	1.10(0.03)	1.23(0.03)	0.002	1.14(0.03)	1.26(0.05)	0.001
IBI	7.46(0.59)	12.75(1.10)	0.001	9.58(0.72)	15.83(1.39)	0.001
AISI	263.30(8.47)	299.73(8.89)	0.001	276.75(6.98)	299.25(8.02)	0.011

MASLD = Metabolic dysfunction–associated steatotic liver disease, BMI = Body mass index, WC = Waist circumference, TG = Triglyceride, TC = Total cholesterol, LDL = Low-density lipoprotein cholesterol, HDL = High-density lipoprotein cholesterol, FPG = Fasting plasma glucose, Hb1Ac= Glycohemoglobin A1c, SII = Systemic immune-inflammation index, SIRI = Systemic inflammation response index, PLR = Platelet-to-lymphocyte ratio, LMR = Lymphocyte-to-monocyte ratio, PNI = Prognostic nutritional index, NLR = Neutrophil-to-lymphocyte ratio, NPAR = Neutrophil percentage to albumin ratio, PIV = Pan-immune-inflammation value, IBI = Inflammatory burden index, AISI = Aggregate index of systemic inflammation.

Supplementary Table 3. Logistic regression models of systemic inflammatory indices with MASH based on model 3

		Q2		Q3		Q4	
Index	Q1	OR (95% CI)	*P*	OR (95% CI)	*P*	OR (95% CI)	*P*
SII	ref	1.165(0.841-1.614)	0.359	1.380(0.995-1.913)	0.054	1.464(1.015-2.113)	0.041
SIRI	ref	1.606(1.107-2.331)	0.013	1.699(1.170-2.467)	0.005	2.040(1.404-2.964)	<0.001
PLR	ref	0.695(0.509-0.949)	0.022	0.643(0.473-0.875)	0.005	0.590(0.420-0.828)	0.002
LMR	ref	1.302(0.934-1.815)	0.122	1.203(0.856-1.692)	0.286	1.543(1.066-2.233)	0.021
PNI	ref	1.483(1.033-2.129)	0.033	1.488(1.049-2.110)	0.026	2.517(1.808-3.505)	<0.001
NLR	ref	0.900(0.663-1.222)	0.500	0.849(0.614-1.173)	0.321	1.442(1.058-1.966)	0.021
NPAR	ref	0.937(0.732-1.199)	0.604	0.920(0.714-1.185)	0.518	0.767(0.574-1.026)	0.074
PIV	ref	0.807(0.557-1.168)	0.255	1.149(0.782-1.688)	0.479	0.933(0.632-1.375)	0.725
IBI	ref	0.920(0.606-1.398)	0.697	1.820(1.203-2.755)	0.005	2.054(1.366-3.088)	0.001
AISI	ref	0.947(0.654-1.371)	0.772	1.010(0.694-1.469)	0.959	1.213(0.828-1.779)	0.322

SII = Systemic immune-inflammation index, SIRI = Systemic inflammation response index, PLR = Platelet-to-lymphocyte ratio, LMR = Lymphocyte-to-monocyte ratio, PNI = Prognostic nutritional index, NLR = Neutrophil-to-lymphocyte ratio, NPAR = Neutrophil percentage to albumin ratio, PIV = Pan-immune-inflammation value, IBI = Inflammatory burden index, AISI = Aggregate index of systemic inflammation.

Supplementary Table 4. Logistic regression models of systemic inflammatory indices with MASLD and significant liver fibrosis based on model 3 (CAP > 288 dB/m; LSM >8 kPa)

		MASLD						Liver fibrosis					
		Q2		Q3		Q4		Q2		Q3		Q4	
Index	Q1	OR (95% CI)	*P*	OR (95% CI)	*P*	OR (95% CI)	*P*	OR (95% CI)	*P*	OR (95% CI)	*P*	OR (95% CI)	*P*
SII	ref	1.100(0.821-1.474)	0.522	1.166(0.857-1.586)	0.329	1.427(1.062-1.917)	0.018	1.225(0.813-1.846)	0.331	1.086(0.692-1.706)	0.719	1.177(0.695-1.994)	0.545
SIRI	ref	1.396(1.015-1.920)	0.039	1.590(1.159-2.180)	0.004	1.608(1.154-2.241)	0.005	1.039(0.618-1.745)	0.885	1.386(0.830-2.315)	0.212	1.664(1.089-2.542)	0.019
PLR	ref	0.802(0.602-1.069)	0.133	0.764(0.571-1.021)	0.069	0.695(0.515-0.939)	0.018	0.650(0.428-0.986)	0.043	0.597(0.386-0.923)	0.021	0.632(0.412-0.970)	0.036
LMR	ref	1.215(0.883-1.672)	0.232	1.314(0.985-1.753)	0.063	1.474(1.079-2.012)	0.015	1.030(0.655-1.620)	0.899	1.278(0.743-2.197)	0.375	1.124(0.669-1.889)	0.659
PNI	ref	1.257(0.918-1.720)	0.153	1.316(0.970-1.785)	0.077	2.091(1.547-2.826)	<0.001	1.515(0.903-2.542)	0.116	1.720(1.106-2.674)	0.016	2.457(1.647-3.665)	<0.001
NLR	ref	0.947(0.710-1.263)	0.711	0.833(0.621-1.119)	0.226	0.728(0.537-0.989)	0.042	1.287(0.863-1.921)	0.216	1.301(0.835-2.027)	0.245	0.783(0.490-1.250)	0.305
NPAR	ref	1.034(0.759-1.408)	0.833	1.022(0.746-1.401)	0.891	0.947(0.698-1.284)	0.724	1.011(0.633-1.614)	0.963	0.924(0.567-1.504)	0.749	1.265(0.768-2.085)	0.356
PIV	ref	0.901(0.655-1.239)	0.521	1.145(0.843-1.557)	0.479	1.046(0.758-1.444)	0.784	1.017(0.566-1.827)	0.956	0.925(0.516-1.656)	0.792	1.638(0.924-2.903)	0.091
IBI	ref	1.070(0.766-1.493)	0.693	1.975(1.427-2.733)	<0.001	2.161(1.548-3.017)	<0.001	0.898(0.510-1.583)	0.711	1.387(0.784-2.454)	0.261	1.669(1.051-2.649)	0.029
AISI	ref	0.839(0.611-1.152)	0.277	1.122(0.817-1.541)	0.478	1.013(0.735-1.396)	0.938	0.917(0.533-1.575)	0.753	0.948(0.569-1.579)	0.837	1.188(0.704-2.006)	0.519

SII = Systemic immune-inflammation index, SIRI = Systemic inflammation response index, PLR = Platelet-to-lymphocyte ratio, LMR = Lymphocyte-to-monocyte ratio, PNI = Prognostic nutritional index, NLR = Neutrophil-to-lymphocyte ratio, NPAR = Neutrophil percentage to albumin ratio, PIV = Pan-immune-inflammation value, IBI = Inflammatory burden index, AISI = Aggregate index of systemic inflammation.

Supplementary Table 5. Performance metrics of systemic inflammatory indices for MASLD and significant liver fibrosis screening

Index	AUROC	Sensitivity	Specificity	95% CI	Positive predictive value	Negative predictive value	Threshold
MASLD							
SII	0.4984	0.2592	0.7631	0.4739-0.5229	0.5396	0.4903	0.5099
SIRI	0.5758	0.4609	0.7733	0.5516-0.5999	0.5578	0.5860	0.4609
LMR	0.4984	0.4116	0.6126	0.4739-0.5230	0.5322	0.4929	0.4995
PNI	0.5654	0.5738	0.5252	0.5411-0.5896	0.5641	0.5351	0.4922
IBI	0.6520	0.7543	0.4854	0.6287-0.6752	0.6108	0.6485	0.4882
Significant liver fibrosis							
SIRI	0.5233	0.4306	0.6294	0.4930-0.5537	0.2503	0.7937	0.2113
PNI	0.5080	0.2542	0.7899	0.4778-0.5382	0.2579	0.7866	0.2154
AISI	0.5363	0.4096	0.6541	0.5066-0.5662	0.2539	0.7941	0.2095
IBI	0.2035	0.6932	0.4918	0.5878-0.6453	0.2815	0.8481	0.2035

SII = Systemic immune-inflammation index, SIRI = Systemic inflammation response index, LMR = Lymphocyte-to-monocyte ratio, PNI = Prognostic nutritional index, IBI = Inflammatory burden index, AISI = Aggregate index of systemic inflammation.

Supplementary Table 6. Threshold Effects of systemic inflammatory indices on MASLD and significant liver fibrosis risk

Variable	Breakpoint	OR_before (95% CI)	OR_after (95% CI)	P_nonlinear
**MASLD**				
SII	193.3624	0.999 (0.997-1.001)	0.999 (0.996-1.002)	0.5500
SIRI	17.3333	1.059 (1.027-1.091)	1.019 (0.988-1.050)	0.2404
PNI	16.5	1.084 (1.024-1.147)	1.022 (0.926-1.128)	0.6676
LMR	5.2	0.983 (0.905-1.066)	1.010 (0.895-1.139)	0.8769
PLR	91.1424	0.966 (0.954-0.979)	0.999 (0.996-1.002)	0.4390
IBI	1.1552	6.790 (2.914-15.819)	1.003 (0.995-1.011)	0.4285
**Significant liver fibrosis**				
SIRI	15.225	1.029 (0.974% CI))f	0.996 (0.976% CI))f	0.7473
PNI	16.5	1.036 (0.948% CI))f	1.001 (0.979% CI)))	0.9057
IBI	6.0064	1.078 (0.965% CI)))	1.003 (0.999% CI)))	0.1606
PLR	112.5	0.989 (0.982% CI)))	0.997 (0.993% CI)))	0.1280
AISI	184.6707	0.995 (0.989% CI)))	1.000 (1.000% CI)))	0.6490

SII = Systemic immune-inflammation index, SIRI = Systemic inflammation response index, LMR = Lymphocyte-to-monocyte ratio, PNI = Prognostic nutritional index, IBI = Inflammatory burden index, AISI = Aggregate index of systemic inflammation.

Supplementary Table 7. Characteristics of participants after propensity score matching

Characteristics	Non- significant liver fibrosis(n=1070)	Significant Liver fibrosis(n=1070)	P-value
Age (year)	52.50(0.99)	52.16(1.18)	0.334
Gender(n,%)			0.069
Male	554(51.78)	596(55.70)	
Female	516(48.22)	474(44.30)	
Race(n,%)			0.995
Mexican American	121(11.31)	122(11.40)	
Other Hispanic	113(10.56)	109(10.19)	
Non-Hispanic white	400(37.38)	277(37.95)	
Non-Hispanic black	299(27.94)	293(27.38)	
Other	137(12.81)	140(13.08)	
Education level(n,%)			0.921
Less than high school	199(18.60)	197(18.41)	
High school or equivalent	252(23.55)	260(24.30)	
Above high school	619(57.85)	613(57.29)	
Marital status (n, %)			0.925
Married/cohabitant	636(59.44)	637(59.53)	
Widowed/divorced/separated	265(24.77)	270(25.23)	
Never married	169(15.79)	163(15.24)	
Poverty income ratio (n, %)			0.969
<1.30	291(27.20)	286(26.73)	
1.30-3.50	427(39.90)	431(40.28)	
>3.50	352(32.90)	353(32.99)	
Drinking status (n, %)			0.965
Non	410(38.32)	411(38.41)	
Low to moderate	660(61.68)	659(61.59)	
Smoking habits (n, %)			0.826
Never	410(38.32)	402 (37.57)	
Moderate	434(40.56)	448(41.87)	
Heavy	226(21.12)	220(20.56)	
Physical activity (n, %)			0.548
Never	278(25.98)	297(27.76)	
Insufficient	139(12.99)	127(11.87)	
Constant	653(61.03)	646(60.37)	
Diabetes(n, %)			0.688
Yes	397(37.10)	406(37.94)	
No	673(62.90)	664(62.06)	
Hypertension(n, %)			0.568
Yes	765(71.50)	753(70.37)	
No	305(28.50)	317(29.63)	

(*Continued*)

Supplementary Table 7. Characteristics of participants after propensity score matching (*Continued*)

Characteristics	Non- significant liver fibrosis(n=1070)	Significant Liver fibrosis(n=1070)	P-value
BMI (kg/m2)			0.617
<28	262(24.49)	272(25.42)	
≥72	808(75.51)	798(74.58)	
SIRI	15.95(0.37)	16.86(0.37)	0.257
PLR	128.15(2.19)	119.71(2.02)	0.008
PNI	14.79(0.18)	15.26(0.16)	0.637
AISI	329.87(9.55)	342.09(10.65)	0.001
IBI	13.15(1.58)	14.20(1.10)	0.349

BMI = Body mass index, SIRI = Systemic inflammation response index, PLR = Platelet-to-lymphocyte ratio, PNI = Prognostic nutritional index, IBI = Inflammatory burden index, AISI = Aggregate index of systemic inflammation.

Supplementary Table 8. Logistic regression models of SIRI, PNI, IBI, PLR, and AISI with significant liver fibrosis after propensity score matching based on model 3

		Q2		Q3		Q4	
Index	Q1	OR (95% CI)	*P*	OR (95% CI)	*P*	OR (95% CI)	*P*
SIRI	ref	1.131(0.781-1.636)	0.515	1.461(0.996-2.143)	0.053	1.744(1.170-2.600)	0.006
PLR	ref	0.711(0.493-1.024)	0.067	0.593(0.416-0.845)	0.004	0.511(0.353-0.741)	<0.001
PNI	ref	1.240(0.859-1.789)	0.250	1.338(0.922-1.940)	0.125	2.043(1.416-2.946)	<0.001
AISI	ref	0.909(0.630-1.312)	0.612	1.361(0.933-1.983)	0.109	1.533(1.064-2.208)	0.022
IBI	ref	1.127(0.637-1.991)	0.682	1.130(0.645-1.981)	0.669	2.196(1.216-3.966)	0.009

SIRI = Systemic inflammation response index, PLR = Platelet-to-lymphocyte ratio, PNI = Prognostic nutritional index, IBI = Inflammatory burden index, AISI = Aggregate index of systemic inflammation.
